# Homology-driven assembly of NOn-redundant protEin sequence sets (NOmESS) for mass spectrometry

**DOI:** 10.1093/bioinformatics/btv756

**Published:** 2015-01-06

**Authors:** Tikira Temu, Matthias Mann, Markus Räschle, Jürgen Cox

**Affiliations:** ^1^Computational Systems Biochemistry and; ^2^Proteomics and Signal Transduction, Max Planck Institute of Biochemistry, Martinsried 82152, Germany

## Abstract

**Summary:** To enable mass spectrometry (MS)-based proteomic studies with poorly characterized organisms, we developed a computational workflow for the homology-driven assembly of a non-redundant reference sequence dataset. In the automated pipeline, translated DNA sequences (e.g. ESTs, RNA deep-sequencing data) are aligned to those of a closely related and fully sequenced organism. Representative sequences are derived from each cluster and joined, resulting in a non-redundant reference set representing the maximal available amino acid sequence information for each protein. We here applied NOmESS to assemble a reference database for the widely used model organism *Xenopus laevis* and demonstrate its use in proteomic applications.

**Availability and implementation:** NOmESS is written in C#. The source code as well as the executables can be downloaded from http://www.biochem.mpg.de/cox. Execution of NOmESS requires BLASTp and cd-hit in addition.

**Contact:** cox@biochem.mpg.de

**Supplementary information:** Supplementary data are available at *Bioinformatics* online.

## 1 Introduction

Within the shotgun proteomics workflow, identification and quantification of proteins relies on comprehensive databases, which are used to calculate theoretical peptide spectra that can be searched against the experimentally determined MS/MS spectra. Therefore, the database should ideally represent the amino acid sequences of all the expressed proteins of the biological system. This information can be deduced from the sequencing of expressed sequence tags (ESTs) as well as from RNA deep-sequencing experiments. These short reads are assembled into larger contigs using genomic sequence information of the same organism as a scaffold. However, for many organisms an accurately assembled genomic sequence is still lacking, leaving investigators with large and redundant sequence repositories that are impractical and of limited use for proteomic studies.

**Fig. 1:**
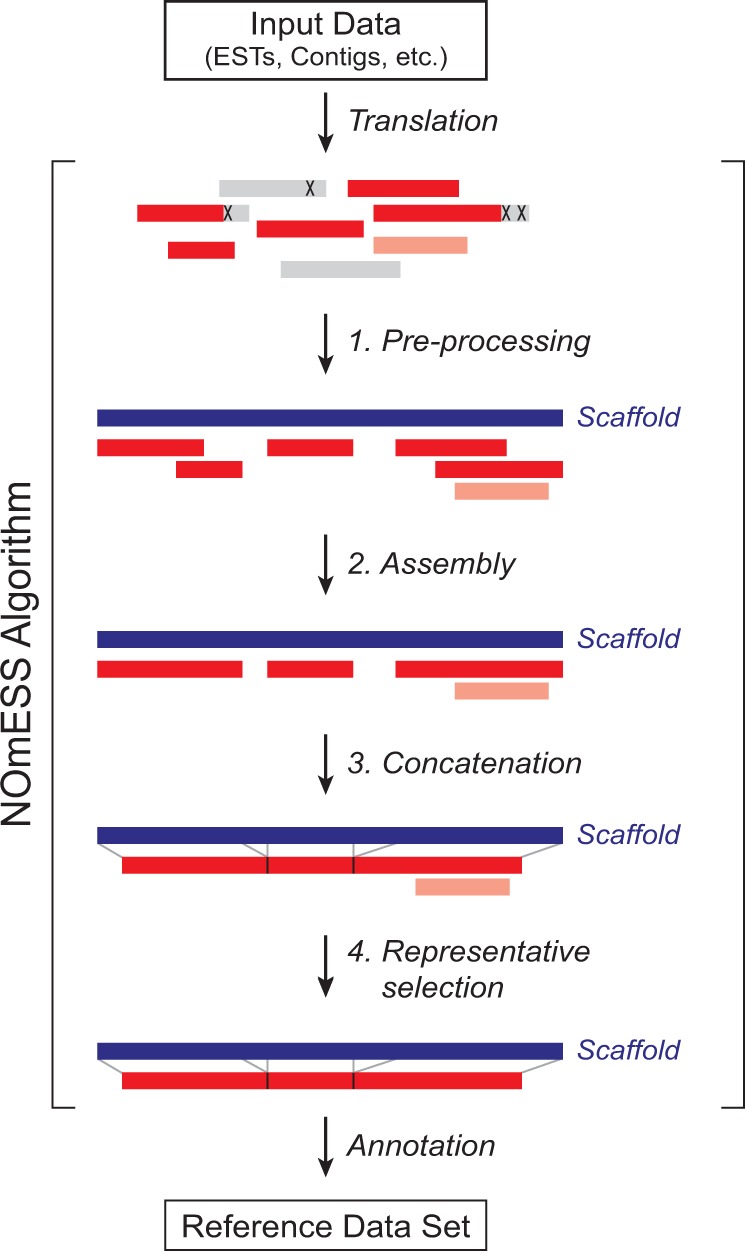
The four basic steps of the NOmESS algorithm

To overcome these limitations, we developed NOmESS, an automated pipeline for the assembly of available sequence information (e.g. ESTs, Contigs, RNAseq, exome sequencing data etc.) into a non-redundant reference dataset based on homology to a closely related species (Fig. 1). As a test organism, we chose *Xenopus* *laevis*, which represents a widely used model organism for developmental and cell cycle studies. Because *X.laevis* underwent allotetraploidization (Hughes and Hughes, 1993), sequencing efforts have been focused predominantly on its diploid cousin *X.tropicalis*, whose complete genome sequence was published in 2010. However, many experiments can only be carried out with *X.laevis* due to its larger size and the availability of species-specific reagents. Using *X.tropicalis* as a scaffold, NOmESS was able to consolidate sequence information from various repositories (e.g. Xenbase, Karpinka *et al.*, 2015), Uniprot, TIGR) into a single non-redundant FASTA file containing about 13 500 protein sequences, which in most cases cover the entire open reading frame of the corresponding *X.tropicalis* homolog.

## 2 Methods

The NOmESS executable and all used sequence sets can be downloaded from http://www.biochem.mpg.de/cox. It requires local installation of BLASTp and cd-hit (Li and Godzik, 2006). A graphical user interface (GUI) (Supplementary Fig. S1) facilitates the uploading of input and scaffold sequences, as well as the setting of all internal parameters. A detailed description of the parameters and their default values is provided in Supplementary Figure S1. FASTA files containing amino acid sequences for both input and scaffold data can be generated with ORF-Predictor (Min *et al.*, 2005). It is recommended to remove redundant entries from the scaffold sequence set by using the implemented cd-hit algorithm using an identity threshold of 0.95, which can be defined in the GUI.

## 3 Results and discussion

The basic steps of NOmESS are shown in Figure 1. It starts with an optional *pre-processing step*, in which input sequences are cut before and after non-amino acid characters (e.g. ‘X’ or '*') and a non-redundant set of fragments with a minimal length of at least 100 amino acids are kept for the assembly process.

In the *assembly step*, input fragments are assigned to the best fit-ting scaffold sequence. To this end, input sequences are blasted against the scaffold sequences and the BLAST hits are filtered by user-defined criteria (see Supplementary Fig. S1 ‘Homology thresholds BLASTp’ tab for default parameters). Fragments corresponding to a scaffold sequence are sorted into a ranking list according to their relative starting position, fragment length and sequence conservation (see Supplementary Fig. S2A and B for details). Starting from the N-terminus, overlapping fragments are identified by matching the end of the first fragment to the subsequent fragment. Only if 9 of the 10 terminal amino acids are identical and if the rest of the overlapping region between the fragments shares at least 97% identity, the C-terminal extension of the second fragment is transferred to the preceding sequence. If this is not the case, the sequence is added at the end of the ranking list. In the rare case that a scaffold sequence is represented by two homologs in the query species, this procedure allows to independently assemble these homologs in a consecutive fashion. Next, redundant sequences are removed from the assembled contigs with cd-hit using an identity threshold of 0.95. In the *concatenation step*, non-overlapping contigs belonging to the same scaffold sequence are fused. To this end, the assembled contigs are realigned to the best-fitting scaffold sequence and sorted by relative position, length and sequence conservation (Supplementary Fig. S2C). Non-overlapping contigs belonging to the same scaffold are then concatenated with a letter ‘X’ separating the two segments to symbolize insertions or deletions. As before, redundant sequences are removed from the concatenated contigs with cd-hit using an identity threshold of 0.95. In the *representative selection step*, the concatenated sequences are blasted against the scaffold sequences and the longest sequence of each cluster is retained to represent the scaffold sequence in the final database. If two sequences have the same length, the BLAST hit with the higher bit score is taken.

NOmESS was developed with the clear intention to facilitate quantitative mass spectrometry based experiments. The goal was to generate a non-redundant reference set, in which each protein is represented by the longest possible amino acid sequence. While the generic approach allows combining heterogeneous data from various sources (e.g. tissues, developmental stages, individuals), concatenation of fragments belonging to different isoforms or allelic variants may result in erroneous hybrid sequences. However, such hybrids may be efficiently removed, as all sequences are filtered stringently after each step for their homology to the scaffold. More importantly, protein intensities are calculated based on the intensity of observed peptides only. Thus, sequence insertion corresponding to an isoform expressed in another tissue than the one analyzed will not affect quantitation of the remaining part of the protein. These occasional draw-backs are outweighed by a general gain in sequence coverage and improved annotation due to the net gain in sequence length.

To demonstrate the usefulness of NOmESS, we used amino acid sequences of *X.tropicalis* as a scaffold to assemble *X.laevis* sequences into a non-redundant reference dataset (Supplementary Fig. S3). We next processed a small MS dataset recorded from fractionated *X.laevis* egg extracts with NOmESS output files generated after different steps of the assembly process. Using these files significantly higher numbers of peptides or ‘protein groups’ were identified compared to identical searches using the latest release from UniProt (Supplementary Fig. S4). Only after the final optional step 4 (‘Representative selection’), the identification rate dropped slightly below the one obtained with the UniProt database. These results suggest that the final step, during which all isoforms are discarded, may be too stringent for discovery-driven projects. However, for more global, pathway-centred analyses, it may greatly facilitate downstream bioinformatic analyses as demonstrated in recent work, in which a NOmESS-generated database has been successfully used to identify *X.laevis* proteins specifically recruited to stalled DNA replication forks (Raschle *et al.*, 2015).

## Supplementary Material

Supplementary Data
