# Supplementary material for: Homology-driven assembly of NOn-redundant protEin sequence sets (NOmESS) for mass spectrometry
Source: Bioinformatics. 2015 Jan 6;32(9):1417–9. doi: 10.1093/bioinformatics/btv756 (PMC4848398; doi:10.1093/bioinformatics/btv756)
Supplement: Supplementary Data [file supp_32_9_1417__index.html]

Homology-driven assembly of NOn-redundant protEin sequence sets (NOmESS) for mass spectrometry — Homology-driven assembly of NOn-redundant protEin sequence sets (NOmESS) for mass spectrometry — Supplementary Data 

# Homology-driven assembly of NOn-redundant protEin sequence sets (NOmESS) for mass spectrometry

## Supplementary Data

files

- Supplementary Data - pdf file
