# Supplementary material for: Homology-driven assembly of NOn-redundant protEin sequence sets (NOmESS) for mass spectrometry
Source: Bioinformatics. 2015 Jan 6;32(9):1417–9. doi: 10.1093/bioinformatics/btv756 (PMC4848398; doi:10.1093/bioinformatics/btv756)
Supplement: Supplementary Data [file supp_btv756_supplementary_material.pdf]

Gene expression

Homology-driven assembly of NOn-redundant protEin Sequence Sets (NOmESS) for mass spectrometry

Tikira Temu[1,2], Matthias Mann[2], Markus Räschle[2*] and Jürgen Cox[1*]
[1]Computational Systems Biochemistry, Max Planck Inst. of Biochemistry, Am Klopferspitz 18, 82152 Martinsried, Germany
[2]Proteomics and Signal Transduction, Max Planck Inst. of Biochemistry, Am Klopferspitz 18, 82152 Martinsried, Germany
Received on XXXXX; revised on XXXXX; accepted on XXXXX
Associate Editor: Janet Kelso

Bioinformatics, XXXXXX

Supplementary Material

This supplement contains:

-Fig. S1-S4

© Oxford University Press 2015

Fig. S1

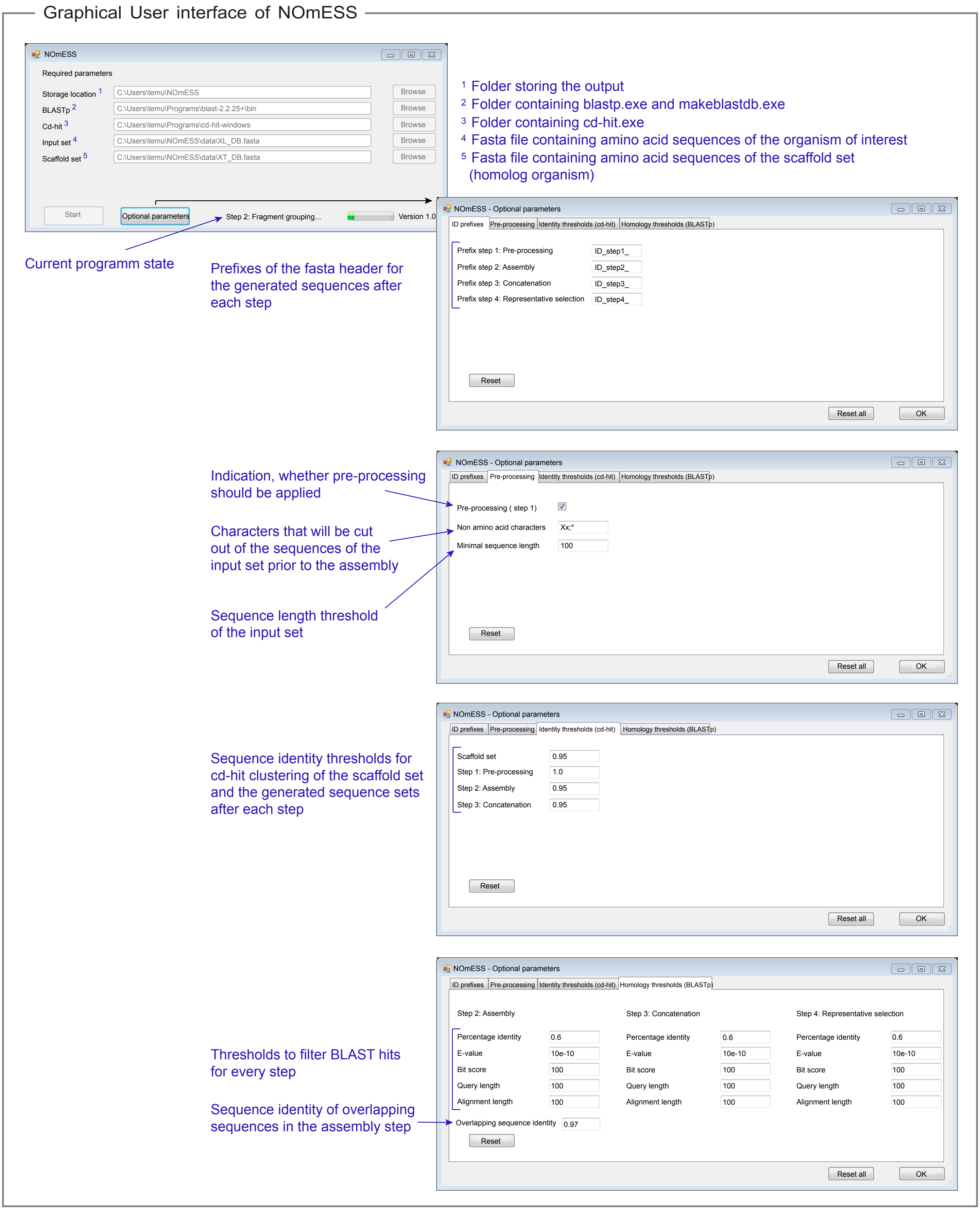

Fig. S1 Graphical User interface of NOmESS. The graphical user interface consists of a main window containing the required parameters, whereas the optional parameters can be found in a pop-up window with 4 tabs. The optional parameter values of the screenshots above are NOmESS' default values. Parameter descriptions are highlighted in blue.

Fig. S2

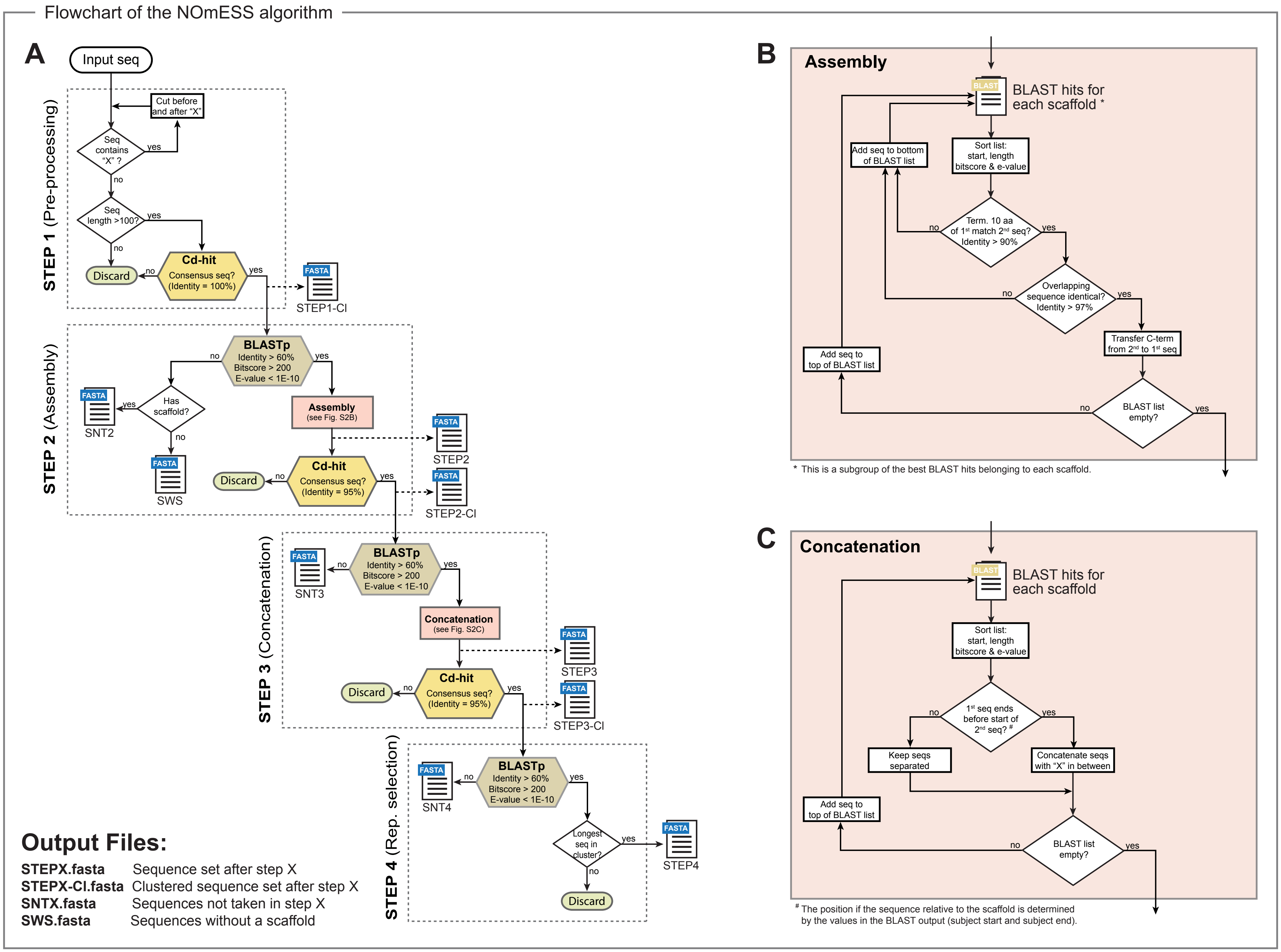

Fig. S2 Flowchart of the NOmESS algorithm. (A) Iterative BLAST searches and cd-hit clustering are used to align sequences to the scaffold, retrieve their relative position and find the representative of each sequence after each step. (B-C) Detailed view of the assembly and the concatenation module (step 2 and step 3 respectively). Abbreviations: seq: sequence, rep.: representative, Term.: terminal, aa: amino acid

Fig. S3

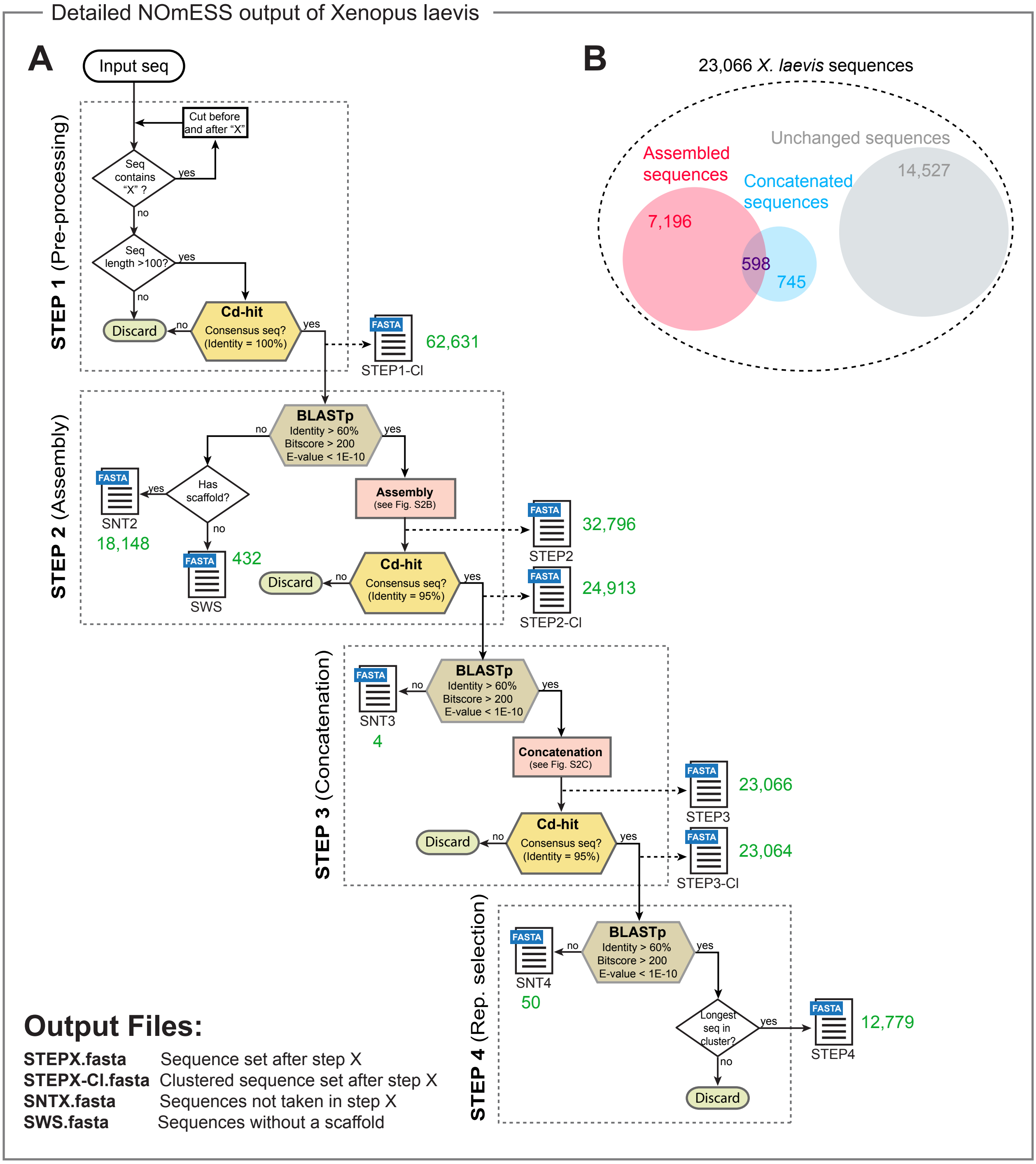

Fig. S3 NOmESS assembly of X. laevis sequences along a X. tropicalis scaffold. (A) Sequences retrieved from various repositories (e.g. TIGR gene indices, Xenbase, contigs from Gurdon, Unigene or XGI, see http://www.biochem.mpg.de/cox) were assembled using amino acid sequences from *X. tropicalis* as a scaffold. The Flowchart indicates the number of sequences generated during each step of the NOmESS procedure (see green numbers next to the output files). (B) Venn diagram showing the number of *X. laevis* sequences that were assembled and/or concatenated. Divergence of *X. laevis* and *X. tropicalis* has been estimated to have occurred about 63.7 million years ago (Evans et. al., 2007).

Fig. S4

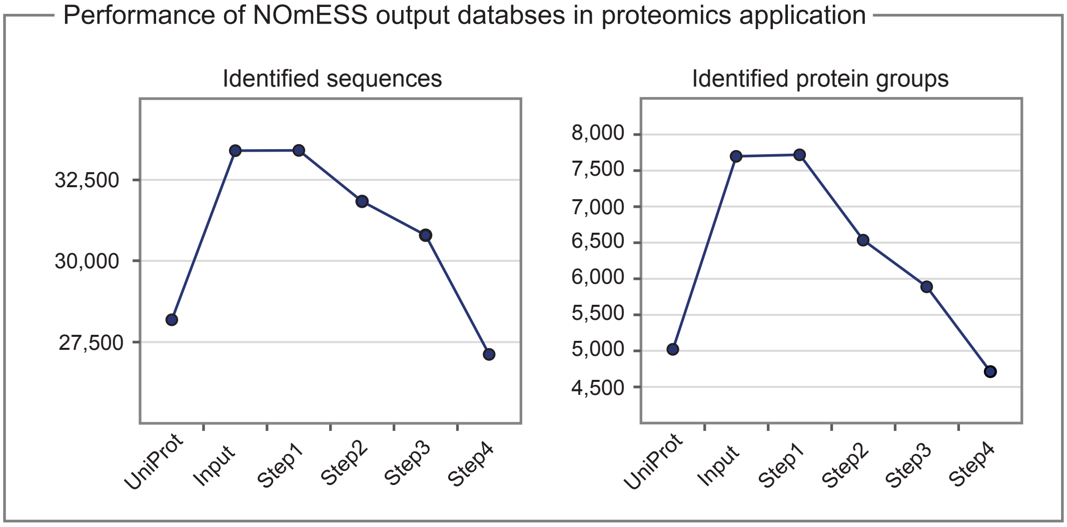

Fig. S4 Comparison of proteomic analyses of a X. laevis egg extracts using NOmESS or UniProt databases. A small mass spectrometry data set acquired from a fractionated egg extract (see Räschle, et al., 2015) was analysed with MaxQuant using the latest available UniProt database or various outputs of the NOmESS pipeline (see Fig. S3). The number of identified peptides (left panel) or “protein groups” (right panel) are plotted for each analysis.
